# A renormalization group analysis of bubble breakup

**DOI:** 10.1038/s41598-025-17667-x

**Published:** 2025-10-03

**Authors:** Ko Okumura

**Affiliations:** Physics Department and Soft Matter Center, Ochanomizu University, 2-1-1 Ohtsuka, Bunkyo-ku, 112-8610 Tokyo, Japan

**Keywords:** Phase transitions and critical phenomena, Fluid dynamics

## Abstract

The self-similarity has been discussed repeatedly for the singular dynamics such as breakup of a fluid drop and its resemblance to critical phenomena in thermodynamic transitions has also been pointed out. Although critical phenomena have been well understood by the renormalization group (RG) theory, the counterpart has not been developed for the breakup problem. Here, we apply an RG analysis developed in mathematics for partial differential equations (PDEs) without noise terms to the bubble breakup, or the formation of a fluid drop surrounded by a more viscous fluid. As a result, we show a wide class of nonlinear and complex PDEs shares the same self-similar solution with a simple PDE that describes the interfacial phenomena, forming the bubble-breakup universality class. We reveal that the experimentally observed self-similar dynamics appear as a stable fixed point of the RG. The framework clarifies that the physical origin of the emergence of the self-similar solution is the invariance of the governing equation under a scale transformation, where the invariance, if not initially exists, could be aquired after the repetition of RG. The present study elucidates that the self-similarity and universality in the hydrodynamic analog emerges as a result of the physics at small scales becoming so important, just as the universality in critical phenomena appears as a result of the physics at large scales becoming so important.

## I. Introduction

Self-similar solutions for PDEs, such as Navier-Stokes equations and Einstein’s equation in general relativity, have attracted considerable attention from researchers in the field such as hydrodynamics, soft and hard condensed matter physics, high-energy physics, cosmology, and applied mathematics^1,2^. In fact, self-similar solutions have been discussed for various phenomena, such as viscous instability^3^, drop coalescence^4–7^ , electro-hydrodynamic spout^8^, fluid jet eruption^9^, flow-induced air entrainment^10–12^, selective withdrawal^13^, and capillary leveling^14,15^. Among them, the breakup of a fluid drop, which happens with a change in topology, has been extensively studied^16–20^ and its similarities with critical phenomena have been discussed especially in the early stage^21–23^. However, quest for a deep analogy with critical phenomena has been premature. This is partly because the exploration into dimensionality and symmetry, which has been known to be important in critical phenomena, has not been enough in the hydrodynamic analog: Experimentally, most examples have been the ones with axisymmetry, and even in examples without axisymmetry [e.g., Ref.^6,7^] the control of symmetry has been limited. Recently, however, the breakup of sheet of air^24^ and then that of an elliptic cone^25^ have been realized experimentally and the self-similarity of the dynamics has been revealed, after a series of studies in a similar system^26,27^. More recently, different kinds of self-similarity in the dynamics has been experimentally revealed and deep analogy with critical phenomena has started being explored^28,29^. Theoretically, given that the RG theory uncovered the essence of critical phenomena^30,31^, a natural attempt may be to develop an RG analysis, which is the focus of the present study.

Here, we develop an RG analysis for the axisymmetric bubble breakup (or formation of a fluid drop in a much more viscous surrounding fluid) in non-confined geometry to provide physical understanding of self-similarity and universality in the problem. Although the problem of the axisymmetric bubble breakup has been well-understood in the framework of the dynamical system description (DSD) (see Ref.^32^ or Ch. 3 of Ref.^17^), which involves a matched asymptotic expansion and stability analysis, the DSD is based on the assumption of the existence of a generalized self-similar solution so that it cannot provide physical origin of the emergence of the self-similarity. On the contrary, our RG analysis, which incorporates the DSD but based on a scale transformation, elucidates that the scale-invariance of the governing equation naturally leads to the self-similar solution, which emerges as a fixed point of the RG, giving a novel view on the DSD: the flow equation in the DSD will be given a new meaning as the RG flow equation. As seen below, the present RG transformation involves magnification of space and the repetition of the RG transformation leads the self-similar solution. This elucidates that the self-similarity emerges as a result of the physics at small scales becoming so important. In addition, our RG analysis proveds a new result that has not been discussed in the DSD: we show that a large class of nonlinear and complex PDEs shares the same self-similar solution with a simple PDE that governs the breakup dynamics, forming a universality class.

**Fig. 1:**
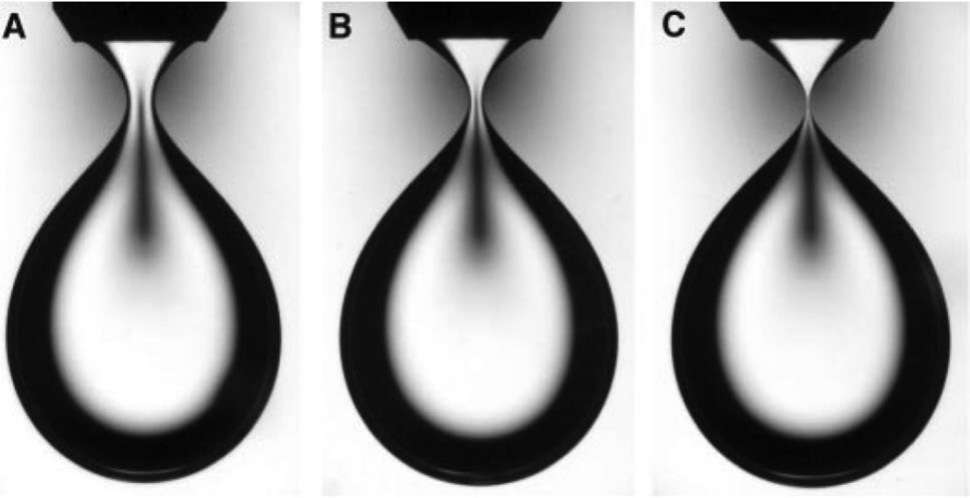
Water drop driping through a highly viscous oil from a nozzle of inner diameter 4.7 mm (Reprinted from Ref.^23^ with permission from AAAS). The constriction region, called neck, thins down with time from A to C, maintaining the same (quadratic) shape.

## II. Formal resemblance between the self-similarity and the scaling hypothesis

We consider the dynamics of the axisymmetric breakup of a bubble (or a fluid drop surrounded by a much more viscous fluid), as shown in Fig. 1. The dynamics can be described by the shape function *h*(*z*, *t*), for which the curve $r=h(z,t)$ represents the liquid-air interface in a cylindrical coordinate $(r,z,\theta )$ with no $\theta$ dependence, where the *r* and *z* coordinates correspond the horizontal and vertical axes. The dynamics is said to be self-similar when the shape at different times is superimposed onto a master curve $\Gamma$ by rescaling:

1 $$\begin{aligned} h(z,t)/r_{0}(t)=\Gamma (z/z_{0}(t)). \end{aligned}$$

When the lengths for rescaling $r_{0}(t)$ and $z_{0}(t)$ exhibit scaling with the time measured from the breakup time $t=t_{c}$, i.e.,

2 $$\begin{aligned} r_{0}(t)\sim \left| t^{\prime }\right| ^{a}\text { and }z_{0} (t)\sim \left| t^{\prime }\right| ^{b}\text { with }t^{\prime }=t-t_{c}, \end{aligned}$$

the self-similarity expressed in Eq. (1) has the same structure with the scaling hypothesis in critical phenomena^30,31^, as explained below.

In fact, in the axisymmetric breakup of a bubble, it is known (see, e.g., Ch. 3.5.1 of Ref.^17^) that $a=1$, $b=1/2$, and

3 $$\begin{aligned} \Gamma (X)=\left\{ \begin{array}{ccc} 1+CX^{2} & & t<t_{c}\\ -1+CX^{2} & & t>t_{c} \end{array} \right. , \end{aligned}$$

where *C* is a constant dependent on the initial conditions. This means $h(z,t)\sim \left| t^{\prime }\right| +Cz^{2}$ for $t<t_{c}$, which shows the parabolic shape translates linearly in time. In other words, the constriction point is always located at $z=0$ and it moves in the $-r$ direction with a velocity *u*. This velocity is known to be given by

4 $$\begin{aligned} u=\gamma /(2\eta ), \end{aligned}$$

as a result of competition between surface tension $\gamma$ and viscosity $\eta$ (see, e.g., Ch. 9.3.1 of Ref.^17^).

In critical phenomena widely observed in nature^30,31^, various physical quantities exhibit power laws $\sim$ $\Delta T^{a'}$ (where $a'$ is an exponent) near the critical temperature $T_{c}$ with $\Delta T=\left| T-T_{c}\right|$. Typical example is the ferromagnetic transition, in which the magnetization *M*(*T*, *H*) is “the order parameter,” a quantity which is zero above $T_{c}$ and nonzero below $T_{c}$ at zero field, i.e., $H=0$. Here, *T* and *H* are dimensionless temperature and magnetic field, respectively. The scaling hypothesis in the critical phenomena for the ferromagnetic transition is expressed as

5 $$\begin{aligned} M(T,H)\simeq \Delta T^{\beta }\Psi (H/\Delta T^{\Delta }), \end{aligned}$$

where $\beta$ and $\Delta$ are called critical exponents and $\Psi (X)$ is the scaling function.

We now explain the correspondence between Eq. (1) with Eq. (2) and Eq. (5). Since *h*(*t*, *z*) is zero after $t_{c}$ and nonzero before $t_{c}$ at $z=0$, this quantity corresponds to the order parameter, where time *t* and position *z* play the role of temperature *T* and external field *H*, respectively: the dynamics before and after breakup respectively correspond to the temperature range below and above $T_{c}$. Furthermore, the master curve represented by $\Gamma (X)$ corresponds to the scaling function $\Psi (X)$, and critical exponents $\beta$ and $\Delta$ in the present case are identified with *a* and *b*, respectively. We remark here for statistical physisists that, although Eq. (1) is a dynamic scaling, i.e., scaling with respect to a time-dependent function *h*(*t*, *z*), this quantity can be identified with the (stationary) order parameter (rather than the time-dependent correlation function) if we regard *t* as a temperature variable.

In critical phenomena, several critical exponents, such as $\alpha$, $\beta$, and $\gamma$ (which should not be confused with the surface tension), are known and a class of substances sharing the same critical exponents is called a universality class. Before the advent of RG, remarkably, a few “scaling relations,” such as $\alpha +2\beta +\gamma =2$, which are simple relations among scaling exponents, are known to be universally satisfied across different classes. Historically, the scaling hypothesis was conjectured to explain scaling relations and the physical origin of the hypothesis is uncovered by the renormalization group theory, which also allows calculation of the value of critical exponents. In other words, the RG solved a longstanding and important problem in the field of physics to explain the physical origin for the scaling hypothesis.

Considering this background, the standard DSD analysis leading to the results summarized in Eq. (1) to (3) may not be fully satisfactory for all physicists, since as explained below the DSD assumes the existence of self-similar solution. In addition, from the close analogy between critical phenomena and its hydrodynamic analogy seen above, we expect an RG analysis could be useful for the hydrodynamic analog. In fact, we show below an RG analysis successfully explains the physical origin of the emergence of self-similarity and elucidate deep connection between the two.

## III. RG analysis for axisymmetric bubble breakup

While there are a number of different types of RG analysis, we exploit below the one developed for PDEs without noise term in applied mathematics^33^, which is quite different from the one by physicists, Goldenfeld and coworkers (see, e.g., Chapter 10 of Ref.^31^), as discussed in Sec. 4. The original study^33^ was on the examination of the long-time diverging solutions of diffusion equations, which corresponds to the case where *L* that appears below is larger than one. This method was applied in the case of $L<1$ for Einstein’s equation in general relativity^34^ and reviewed in Ref.^35^ in the case of $L>1$. In the following, we apply the case of $L<1$ with incorporating the DSD analysis. Accordingly, to provide a coherent description, we also explain the content of the DSD (Sec. 3F to 3H), following the arguments in Ref.^32^ or Chapter 3 of Ref.^17^. On the contrary, the descriptions in Sec. 3B to 3E are original arguments on the application of the RG analysis developed in Ref.^33^ but in the case $L<1.$

### A. Equation of motion for the interface

We consider the breakup of a bubble as in Fig. 1 as before. From the axisymmetry, the $\theta$ component of the velocity field of the viscous fluid is zero, while the $\theta$-independent *r* and *z* components are denoted *u*(*t*, *r*, *z*) and *v*(*t*, *r*, *z*), respectively. When a point on the interface (*r*, *z*) with $r=h(t,z)$ translates with a velocity

6 $$\begin{aligned} (u_{s}(t,z),v_{s}(t,z))\equiv (u(t,r=h(t,z),z),v(t,r=h(t,z),z)), \end{aligned}$$

the point moves to another point $(r^{\prime },z^{\prime })=(r+u_{s} dt,z+v_{s}dz)$ with $r^{\prime }=h(t+dt,z^{\prime })$ after *dt*, from which we have the equation of motion of the interface

7 $$\begin{aligned} \frac{\partial h}{\partial t}+v_{s}\frac{\partial h}{\partial z}=u_{s}. \end{aligned}$$

In the bubble breakup, as indicated by Eq. (4), $(u_{s} ,v_{s})=(-\gamma /(2\eta ),0)$, so that Eq. (7) reduces to the following form:

8 $$\begin{aligned} \frac{\partial h}{\partial t}=-\gamma /(2\eta )\text {.} \end{aligned}$$

We stress the relation ($(u_s, v_s)=(-\gamma/(2\eta), 0)$ with Eq. (7) means that the interface $r=h(t,z)$ near the constriction point is just translating in time in the *r* direction without changing the form since $u_{s}$ and $v_{s}$ are coordinate independent.

### B. Scale transformation and a wide class of PDEs containig the bubble-breakup universality class

In the following, we consider a wide class of PDEs, which is expressed as Eq. (8) plus terms represented by the function $g(h,h^{\prime },h^{\prime \prime },..)$ where $h^{\prime }=\partial h(z,t)/\partial z$, $h^{\prime \prime }=\partial ^{2}h(z,t)/\partial z^{2}$,...:

9 $$\begin{aligned} \frac{\partial h(z,t)}{\partial t}=-\gamma /(2\eta )-g(h,h^{\prime } ,h^{\prime \prime },..). \end{aligned}$$

The function $g(x,y,z,..)$ that defines the extra terms could be very complex and nonlinear: we only assume that $g$ is analytic in a neighborhood of the origin, i.e., $g(h,h^{\prime },h^{\prime \prime },..)$ could be the sum of nonlinear and complex monomial function such as $\left[ h(z,t)\right] ^{l}\left[ \partial h(z,t)/\partial z\right] ^{n}\left[ \partial ^{2}h(z,t)/\partial z^{2}\right] ^{m}$. As shown below, a wide class of nonlinear PDEs described by Eq. (9) shares the same self-similar solution with the simple PDE in Eq. (8) and thus forms a universality class, if $g$ satisfies a positivity condition specified below.

We introduce dimensionless variables by $H=h/h_{0}$, $T=(t_{c}-t)/(t_{c} -t_{0})$, and $X=(z-z_{c})/h_{0}$ with $h_{0}/(t_{c}-t_{0})=\gamma /(2\eta )$ to express Eq. (9) as

10 $$\begin{aligned} \frac{\partial H(X,T)}{\partial T}=1+G(H,H^{\prime },H^{\prime \prime },...) \end{aligned}$$

with $H^{\prime }=\partial H(X,T)/\partial X$, $H^{\prime \prime }=\partial ^{2}H(X,T)/\partial X^{2}$,..., where $t=t_{0}$ means $T=1$ and $T>0$ for the before-breakup $(t<t_{c})$ dynamics of our focus (note the difference from the after-breakup dynamics where *T* ($>0$) is defined not as $T\sim t_{c}-t$ but as $T\sim t-t_{c}$).

We introduce the following scale transformation

11 $$\begin{aligned} X^{\prime }&=X/L,\,T^{\prime }=T/L^{B} \end{aligned}$$

12 $$\begin{aligned} H^{\prime }(X^{\prime },T^{\prime })&=L^{A}H(X,T)\,\equiv H_{L}(X^{\prime },T^{\prime }) \end{aligned}$$

13 $$\begin{aligned}&\Leftrightarrow \,H_{L}(X,T)=L^{A}H(LX,L^{B}T). \end{aligned}$$

This transformation changes Eq. (10) into the following form:

14 $$\begin{aligned} \frac{\partial H_{L}(X,T)}{\partial T}=L^{A+B}+G_{L}(H_{L},H_{L}^{\prime },H_{L}^{\prime \prime },...), \end{aligned}$$

where

15 $$\begin{aligned} G_{L}(H_{L},H_{L}^{\prime },H_{L}^{\prime \prime },...)=L^{A+B}G(L^{-A}H_{L} ,L^{-A-1}H_{L}^{\prime },L^{-A-2}H_{L}^{\prime \prime },...). \end{aligned}$$

Equation (14) is derived as follows. The replacement $X\rightarrow LX$ and $T\rightarrow L^{B}T$ in the left-hand side of Eq. (10), followed by the use of Eq. (13), results in

16 $$\begin{aligned} \frac{\partial H(LX,L^{B}T)}{\partial L^{B}T}=\frac{\partial H_{L}(X,T)/L^{A} }{L^{B}\partial T}=L^{-A-B}\frac{\partial H_{L}(X,T)}{\partial T}, \end{aligned}$$

which leads to the left-had side of Eq. (14) divided by the factor $L^{A+B}$. To explain Eq. (15), we take a case of a monomial $G(x,y,z,...)=x^{l} y^{n}z^{m}w^{k}\cdots$:

17 $$\begin{aligned} G(H,H^{\prime },H^{\prime \prime },...)=H(X,T)^{l}\left[ \frac{\partial H(X,T)}{\partial X}\right] ^{n}\left[ \frac{\partial ^{2}H(X,T)}{\partial X^{2}}\right] ^{m}\cdots \end{aligned}$$

Under the replacement $X\rightarrow LX$ and $T\rightarrow L^{B}T$, followed by the use of Eq. (13), the term transforms as

18 $$\begin{aligned}&H(LX,L^{B}T)^{l}\left[ \frac{\partial H(LX,L^{B}T)}{\partial (LX)}\right] ^{n}\left[ \frac{\partial ^{2}H(LX,L^{B}T)}{\partial (LX)^{2}}\right] ^{m}\cdots \end{aligned}$$

19 $$\begin{aligned}&=L^{n_{G}}H_{L}(X,T)^l\left[ \frac{\partial H_{L}(X,T)}{\partial X}\right] ^{n}\left[ \frac{\partial ^{2}H_{L}(X,T)}{\partial X^{2}}\right] ^{m} \cdots \end{aligned}$$

with

20 $$\begin{aligned} n_{G}=-(l+n+m+k+\cdots )A-(n+2m+3k+\cdots ) \end{aligned}$$

With the aid of this monomial example, we understand Eq. (15) for the general case, which complete the derivation of Eq. (14).

For later discussion on the “universality across different models” in analogy with critical phenomena, we express Eq. (15) by

21 $$\begin{aligned} G_{L}(H_{L},H_{L}^{\prime },H_{L}^{\prime \prime },...)=L^{A+B}L^{n_{G}}\tilde{G}(H_{L} ,H_{L}^{\prime },H_{L}^{\prime \prime },...), \end{aligned}$$

where $n_{G}$ is defined for an analytic *G* by taking the smallest of the numbers in Eq. (20) computed for the monomials in the Taylor series of *G* at the origin with non-vanishing coefficients, and $\tilde{G}$ is a function which becomes an *L*-independent function of *H*_*L*_, *H'*_*L*_,... in the small *L* limit. In other words, Eq. (10) is transformed into the following PDE:

22 $$\begin{aligned} \frac{\partial H_{L}(X,T)}{\partial T}=L^{A+B}(1+L^{n_{G}}\tilde{G}(H_{L} ,H_{L}^{\prime },H_{L}^{\prime \prime },...)) \end{aligned}$$

We return to this equation in Sec. 3D.

In the following, we consider the scale transformation in which the governing equation of the bubble breakup in Eq. (8), or Eq. (10) with $G=0$, becomes invariant under the scale transformation, i.e., we assume

23 $$\begin{aligned} B=-A\text {,} \end{aligned}$$

which guarantees that Eq. (10) with $G=0$ is transformed into

24 $$\begin{aligned} \frac{\partial H_{L}(X,T)}{\partial T}=1. \end{aligned}$$

### C. RG transformation

In our RG transformation, we are interested in the case of

25 $$\begin{aligned} L<1\text { and }B>0 \end{aligned}$$

for the scale transformation defined in Eq. (11) to (13): the scale transformation corresponds to magnification of space-time, i.e., $X^{\prime }>X$ and $T^{\prime }>T$. This is in contrast with the case of critical phenomena, where the scale transformation corresponding to spacial coarse-graining is important. In the present case, we are interested in gradually going closer to smaller scales in time and space near the singularity at breakup, i.e., $z=z_{c}$ and $t=t_{c}$.

We define a renormalization group (RG) transformation by

26 $$\begin{aligned} R _{L,G}f(X)\equiv L^{A}H(LX,L^{B})=H_{L}(X,1), \end{aligned}$$

where *f*(*X*) gives the initial condition at $T=1$ for *H*(*X*, *T*), i.e., $H(X,1)=f(X)$. Note that the present RG can be regarded as two steps as in critical phenomena, where coarse-graining followed by rescaling. (1) The time evolution based on Eq. (10) (thus $R_{L,G}$ depends on $G$) to a smaller time, which is $L^{B}(<1)$ times smaller: $f(X)=H(X,1)\rightarrow H(X,L^{B})$. This is followed by (2) a rescaling of *X* and *H*: $H(X,L^{B})\rightarrow L^{A}H(LX,L^{B})$. The time evolution to a smaller time towards the critical time $T=0$ or $t=t_{c}$ corresponds to the coarse-graining, or the momentum shell integration of Wilson’s RG for critical phenomena, by which the effective temperature gets closer to the critical temperature.

We define the fixed point of the RG group by

27 $$\begin{aligned} R _{L,G^{*}}f^{*}(X)=f^{*}(X) \Leftrightarrow L^{A}H^{*}(LX,L^{B})=f^{*}(X), \end{aligned}$$

which is a point in the space of function. Here, $G^{*}$ is "scale-invariant" [i.e., invariant under the transformation in Eqs. (11) to (13) with Eq. (23)] as further specified below. If such a point exists, by setting $T=L^{B}$, we obtain $T^{A/B}H^{*}(T^{1/B}X,T)=f^{*}(X)$, or a self-similar solution

28 $$\begin{aligned} H^{*}(X,T)=T^{-A/B}f^{*}(X/T^{1/B}), \end{aligned}$$

which leads to

29 $$\begin{aligned} H^{*}(X,T)=Tf^{*}(\xi ) \end{aligned}$$

with $\xi =X/T^{1/B}$, where $f^{*}(\xi )$ describes the master curve representing the self-similar solution. In other words, *the self-similar solution here is not an assumption but naturally comes out from our renormalization group as the fixed point.*

In general, the initial “point” *f*(*X*), which could be the shape function at any time away from $t=t_{c}$, is not a fixed point. However, if a singularity is approached, we expect the shape becomes universal or self-similar. This corresponds to expecting that after iterating the RG transformation towards $t=t_{c}$, i.e.,

30 $$\begin{aligned} R _{L^{n} }f(X)=R _{L,G_{L^{n-1}}}\circ \cdots\circ R _{L,G_L} \circ R _{L,G}f(X), \end{aligned}$$

the resulting function $R _{L^{n}}f(X)$ flows into the fixed point $f^{*}(X)$, while $G _{L^{n-1}}$ flows into a scale-invariant function $G^{*}$: the fixed point $f^{*}$ introduced above corresponds to Eq. (10) with $G=G^{*}$, which is scale-invariant by virtue of Eq. (23) (otherwise the fixed point could not exist). In other words, we iterate RG with an expectation that the shape function will flow towards *a fixed point in the space spanned by functions*, which leads to the self-similar solution reflecting the scale-invariance of the governing equation, Eq. (10) with $G=G^{*}$. Note that the dependence of $R _{L,G_L}$ on $G_L$ in Eq. (30) reflects that the time evolution of $H_L$ [see Eq. (26)] is described by Eq. (14) with Eq. (23). To be precise, we further remark that (1) after $m$-th iterations the original function $f(X)=H(X,1)$ is transformed into $H_{L^m}(X,1)$, which is followed by the $(m+1)$-th operation $R_{L,G_{L^m}}H_{L^m}(X,1)=L^A H_{L^m}(LX, L^B)$ where the time evolution from $1$ to $L^B$ is based on $G_{L^m}$. This means at the fixed point $H_{L^m}(X,1)=H_{L^{m+1}}(X,1)$, which implies $G_{L^{m-1}}=G_{L^m}$ and Eq. (23) are required: the scale-invariance is the key for the existence of a fixed point and thus of a self-similar solution.

### D. Universality beyond “models”

We now return to Eq. (22) for the discussion on the “universality across different models” in analogy with critical phenomena. From this equation, we see that the extra term $L^{A+B}L^{n_{G}}\tilde{G}(H_{L},H_{L}^{\prime },H_{L}^{\prime \prime },...)$ becomes negligible after iteration of the RG transformation, as far as the exponent $A+B+n_{G}$ is positive, which specifically means that $2l+n-k\cdots$ is positive in the ensuing case, in which $A+B=0$ and, as seen below, $A=-2$. This means, if $g$ or *G* satisfies this positivity condition, the term is irrelevant and the ensuing RG analysis, in which we assume $G=0$ (and thus $G^{*}=0$), is unchanged if $2l+n-k\cdots$ is positive: a large class of PDEs in Eq. (9) or (10) satisfying the positivity condition flows into the same fixed point described by Eq. (8), and thus forms the bubble-breakup universality class. This corresponds to universality across different “models” in critical phenomena, where different “models” correspond to the governing equations with different extra terms.

### E. RG flow equation

In the following, we consider the case of$\:G=0$, as announced. This is because we are interested in the self-similar solution of the bubble-breakup universality class.

In critical phenomena, the corresponding flow with iteration of the RG transformation and its behavior near the fixed point becomes very important. To know the details, the RG flow equation is derived based on the RG transformation, which could involve the momentum-shell integration based on a diagrammatic technique. Compared with this, the derivation of the flow equation in the present case is very simple as seen below.

To find fixed points and the flow equation, we introduce “logarithmic time variable” $\tau$, which will turn out to describe the RG flow*: *$\tau =-\log T=-B\log L$, where $\tau >0$ and $\tau \rightarrow \infty$ as $t\rightarrow t_{c}$ with $0<T<1$. As a result, we obtain

31 $$\begin{aligned}&-B\frac{dH_{L}(X,T)}{d\tau } =L\frac{dH_{L}(X,T)}{dL}&\end{aligned}$$

32 $$\begin{aligned}&\quad =AH_{L}(X,T)+X\frac{\partial H_{L}(X,T)}{\partial X}+BT\frac{\partial H_{L}(X,T)}{\partial T},&\end{aligned}$$

which can be proven by using Eq. (13). When combined with Eq. (24), this equation gives

33 $$\begin{aligned} -B\frac{dH_{L}(X,T)}{d\tau }=AH_{L}(X,T)+X\frac{\partial H_{L}(X,T)}{\partial X}+BT. \end{aligned}$$

By setting $T=1$ in this equation with noting $H_{L}(X,1)=R _{L}f(X)\equiv \widehat{f}(X,\tau )$, we have:

34 $$\begin{aligned} -B\frac{d\widehat{f}(X,\tau )}{d\tau }=-B[\widehat{f}(X,\tau )-1]+X\frac{\partial \widehat{f}(X,\tau )}{\partial X}. \end{aligned}$$

This equation corresponds to the RG flow equation in critical phenomena.

In the DSD^17,32^, the same flow equation in Eq. (34) is derived without discussing the scale transformation (Eq. (11) to (13)), the scale invariance of the bubble-breakup equation (Eq. (24)), and the renormalization group transformation (Eq. (27)). Instead, it is directly derived first by introducing a logarithmic time variable $\tau$ by $\tau =-\log T$, and then substituting *a generalized self-similar form* $H(X,T)=T^{\alpha }\widehat{f}(\xi ,\tau )$ with $\xi =X/T^{\beta }$ into the governing equation in Eq. (10) with $G=0$, where $\alpha$ and $\beta$ correspond to $-A/B$ and 1/*B* in the above. In other words, the self-similarity is a hypothesis in the DSD.

### F. Fixed-point solutions

From the RG flow equation, fixed points are given by the vanishing of the left-hand side since a change in $\tau$ corresponds to a change in *L*: The fixed point in a functional space, $\widehat{f}^{*}(X,\tau )=f^{*}(X)$, can be obtained from the following equation:

35 $$\begin{aligned} -B[f^{*}(X)-1]+X\frac{\partial f^{*}(X)}{\partial X}=0, \end{aligned}$$

which results in

36 $$\begin{aligned} f^{*}(X)=1+CX^{B} \end{aligned}$$

The self-similar solution in Eq. (28) with the fixed-point solution to the RG flow equation in Eq. (36), which includes unknown constants *B* and *C*, should correspond to the observed solution in Eq. (1) to (3). To find conditions for *C* and *B* ($>0$ from Eq. (25)), we impose (i) a matching condition for $T\rightarrow 0$ and (ii) a regularity condition. The first condition $T^{\alpha }f^{*}(X/T^{\beta })=H^{*}(X,T)$ for $T\rightarrow 0$ with $\alpha =-A/B=1$ and $\beta =1/B$ can be translated into the condition for $\left| \xi \right| =\left| X/T^{\beta }\right| \rightarrow \infty$ since $\beta >0$: $f^{*}(\xi )=H^{*}(X,0)(\xi /X)^{\alpha /\beta }$, i.e.,

37 $$\begin{aligned} f^{*}(\xi )\sim \xi ^{\alpha /\beta }\text { as }\left| \xi \right| \rightarrow \infty \end{aligned}$$

This condition, in this case $f^{*}(\xi )\sim \xi ^{B}$, is automatically satisfied in Eq. (36) if $C\ne 0$, meaning that *C* is nonzero. The second condition demands that Eq. (36) be regular for all real *X*, from which *B* ($>0$) should be a positive integer, otherwise Eq. (36) would have a singularity for $X=0$. In addition, we demand the condition $f^{*}(X)\ne 0$ for nonzero real *X* to ensure there are no breakup singularity ($H(X,T)=0$) other than $X=0$. This condition is equivalent to $X=(-1/C)^{1/B}$ (where $C\ne 0$ and *B* is a positive integer) is not a real number, which means $C>0$ and $e^{(2m+1)\pi i/B}$ with *m* zero or integer is not a real number for any *m*. From the last condition, a positive integer *B* should be even:

38 $$\begin{aligned} B=2(i+1)\text { with}\ i=0,1,2,\ldots , \end{aligned}$$

where the case of $i=0$ will be called the ground state and the cases of $i=1,2,,\ldots$ will be called excited states in analogy with quantum mechanics.

### G. Stability analysis

To understand which *i* corresponds to the experimentally observed self-similar solution, we need to examine the stability of the fixed-point solutions by seeking a solution of the RG flow equation in the following form:

39 $$\begin{aligned} \widehat{f}(X,\tau )=f^{*}(X)+\delta f(X,\tau )\text { with }\delta f(X,\tau )=\delta (X)e^{\omega \tau }. \end{aligned}$$

In the present case, as seen below, this stability analysis can be performed explicitly, which shows only the ground state solution

40 $$\begin{aligned} f^{*}(X)=1+CX^{2} \end{aligned}$$

is stable: even if the perturbation $\delta f(X,\tau )$ is initially a superposition of all modes (i.e., $\delta f(X,\tau )=\sum _{k}c_{k}\delta _{k}(X)e^{\omega _{k}\tau }$) with allowed values of $\omega =\omega _{k}$, they disappear (they are, thus, “irrelevant”) and thus the solution flows into the fixed point, which is stable and thus observed in experiment.

*In other words, by virtue of the stability analysis given below, we can fully determine the exponents as *$B=-A=2$* in the self-similar solution in Eq.* (28):

41 $$\begin{aligned} H^{*}(X,T)/T=f^{*}(X/T^{1/2}) \end{aligned}$$

with Eq. (40), i.e., $H^{*}(X,T)$ at different *T* can be collapsed by the rescaling $H^{*}(X,T)/T$ and $X/T^{1/2}$. This result can be written more explicitly as $H^{*}(X,T)=T+CX^{2}$ in the present case, which shows the parabolic shape translates linearly in time. These results are fully consistent with Eq. (1) to (3).

Details of the stability analysis are as follows. Substituting Eq. (39) into the flow equation in Eq. (34), we obtain

42 $$\begin{aligned} B(1-\omega )\delta =Xd\delta /dX \end{aligned}$$

with $B=2(i+1),$ from which we get

43 $$\begin{aligned} \delta (X)\simeq X^{B(1-\omega )}, \end{aligned}$$

where $B(1-\omega )$ should be $j=0,1,2,\ldots$ from the regularity at $X=0$, which means

44 $$\begin{aligned} \omega =1-j/2(i+1)\text { with }\delta (X)=X^{j}. \end{aligned}$$

This results in

45 $$\begin{aligned} i&=0:\omega =1,1/2,0,-1/2,-1,\ldots \end{aligned}$$

46 $$\begin{aligned} i&=1:\omega =1,3/4,1/2,1/4,0,-1/4,\ldots \end{aligned}$$

47 $$\begin{aligned} i&=2:\omega =1,5/6,2/3,1/2,1/3,1/6,0,-1/6,\ldots \end{aligned}$$

and so on, where . . . corresponds to negative numbers.

The negative modes ($\delta e^{\omega \tau }$ with a negative $\omega$) are stable or irrelevant: they tend to disappear and flow into the fixed point since $\tau$ $(>0)$ becomes large as $t\longrightarrow t_{c}$. On the other hand, the positive modes are unstable or relevant: they tend to grow and flow the solution away from the fixed point, while $\omega =0$ is called a marginal mode. However, in fact, as explained in the next section, among the non-negative modes, the following three modes (with “the eigen function” $\delta (X)$ in analogy with quantum mechanics) for a fixed *i* are “apparently unstable (or marginal)” and do not represent truly unstable (or marginal) modes:

48 $$\begin{aligned} j&=0:\omega =1\text { and }\delta (X)\simeq 1 \end{aligned}$$

49 $$\begin{aligned} j&=2i+1:\omega =1/B=1/(2i+2)\text { and }\delta (X)\simeq X^{2i+1} \end{aligned}$$

50 $$\begin{aligned} j&=2i+2:\omega =0\text { and }\delta (X)\simeq X^{2i+2}. \end{aligned}$$

With this understanding, we see for the ground state solution the first three non-negative modes $\omega =1,1/2$, and 0 in Eq. (45) correspond to the special three modes in Eqs. (48) to (50) with $i=0$, which do not represent unstable (or marginal) modes, and the remaining modes are all negative. Thus, the ground state solution in Eq. (40) is stable. However, for the excited states there always remain a finite number of positive modes representing truly unstable modes: for example, for the first excited states ($i=1$), although the modes $\omega =1,1/4,$ and 0 can be removed from the modes listed in Eq. (46) as apparently unstable or marginal modes, there remain positive modes $\omega =3/4$ and 1/2, representing unstable modes. In other words, only for the ground state solution, all the modes around the fixed point is irrelevant and thus the fixed point is an atractor of RG transformation to be observed in experiments.

### H. Apparent modes

In the above stability analysis, we seek different solutions to the flow equation, which amounts to seek different solutions to the governing equation in Eq. (10) with $G=0$. This equation is invariant under translation in time and space. In addition, since the fixed point solution in Eq. (36) contains a constant *C*, and the solution with a shifted *C* is also a solution. The solutions corresponding to these shifts in time, space, and *C* should be included in the mode $\omega$ derived in the previous section, which are, as we will see, the three modes for a fixed $i$ announced in Eq. (48) to (50). These modes do not represent unstable modes. This is because the appearance of such modes represents an inappropriate choice of $t_{c}$, $z_{c}$, and/or *C*, which merely results in non-approaching to the singular point.

To see this scenario explicitly, in the case of a shift in time $t_{c} \longrightarrow t_{c}+\Delta t$, we consider the following expansion up to the leading order in $\Delta t$:

51 $$\begin{aligned} h^{*}(x,t)_{t_{c}\longrightarrow t_{c}+\Delta t}=h^{*}(x,t)_{t_{c} }+\Delta t[\partial h^{*}/\partial t]_{t=t_{c}}. \end{aligned}$$

By re-expressing Eq. (28) with (36) and (38) as $h^{*}(x,t)\simeq T^{\alpha }f^{*}(\xi =X/T^{\beta })$ with $\alpha =-A/B$ and $\beta =1/B$ for notational convenience, where

52 $$\begin{aligned} \alpha =1,\beta =1/(2i+2)\text {, and }f(\xi )=1+C\xi ^{2(i+1)}, \end{aligned}$$

and by remarking $X\simeq z-z_{c}$ and $T\simeq t_{c}-t$, we have

53 $$\begin{aligned} [\partial h^{*}/\partial t]_{t=t_{c}}&\simeq \alpha T^{\alpha -1}f+T^{\alpha }(df^{*}/d\xi )(-\beta \xi /T) \end{aligned}$$

54 $$\begin{aligned}&=T^{\alpha }e^{\tau }(\alpha f-\beta \xi f^{\prime }). \end{aligned}$$

where we use $T^{-1}=e^{\tau }$. This equation with $f(\xi )\simeq T^{-\alpha }h(x,t)$ leads to

55 $$\begin{aligned} \delta f=f^{*}(\xi )_{t_{c}\longrightarrow t_{c}+\Delta t}-f(\xi )_{t_{c} }^{*}\simeq e^{\tau }(\alpha f-\beta \xi f^{\prime })\Delta t, \end{aligned}$$

from which by use of Eq. (52) we obtain : $\delta f\simeq e^{\tau }\Delta t$. This case just corresponds to $\delta f(X,\tau )=\delta (X)e^{\omega \tau }$ in Eq. (39) with $\omega =1$ and $\delta(X) =1$ as specified in Eq. (48). The mode $\omega =1$ appearing in Eq. (44) is now identified with the solution originating from a shift in $t_{c}$ and thus does not represent an unstable mode.

In the case of the translation in space, we obtain $\delta f=$ $f(\xi )_{z_{c}\longrightarrow z_{c}+\Delta z}-f(\xi )_{z_{c}}^{*}$ $\simeq e^{\beta \tau }f^{\prime }\Delta z$, from which we obtain $\delta f\simeq e^{\tau /B}\xi ^{2i+1}\Delta z$ in a similar manner: the mode $\omega =1/B=1/(2i+2)$ appears in Eq. (44), which is specified in Eq. (49), corresponds to the solution originating from a shift in $z_{c}$ and thus does not represent an unstable mode.

For the transformation $C\longrightarrow C+\Delta C$, we clearly have $\delta f=$ $f(\xi )_{C\longrightarrow C+\Delta C}-f(\xi )_{C}^{*}\simeq \Delta C\partial f/\partial C$. From this we obtain $\delta f\simeq \Delta C\xi ^{2i+2}$ as before, which just corresponds to Eq. (39) with $\omega =0$, as specified in Eq. (50). The mode $\omega =0$ appears in Eq. (44) corresponds to the solution originating from a shift in *C* and thus does not represent a marginal mode.

As seen above the three non-negative modes for a fixed *i* in the stability analysis, given in Eq. (48) to (50), do not represent unstable or marginal modes. This reflects the invariance of the governing equation for the three translations.

## IV. On various types of RG

The RG for PDEs in the absence of noise (different from the Langevin or KPZ equation for which functional representation is possible) was initiated in 1990^36^ in a field-theoretical manner due to Gell-Mann and Low^37^ to examine the long-time behavior of a nonlinear diffusion equation^31^. Another type of RG for PDEs in the absence of noise was independently developed by Bricmont, Kupiainen, and Lin (BKL) in the field of applied mathematics in 1994^33^ in a manner more in parallel with the RG due to Wilson^38^ (although there are differences as summarized in Discussion), which is reviewed and developed in Ref.^35^. This BKL version of RG may be less known to physicists and are also for long time behavior, while the BKL version of RG for a singular dynamics near a critical point is developed in Ref.^34^ in the context of cosmology^39^. It is interesting that, in the original studies of the two methods, field-theoretic RG for PDEs^36^ and the BKL RG for PDE^33^, both groups of authors wrote that they were motivated by the same study by Barenblatt^40^. Independently from and earlier than these two approaches, another method (DSD) for the singular dynamics near a singular point of PDEs was developed in applied mathematics by Giga and Kohn in 1985^41^, which is applied for the bubble breakup in Ref.^17^.

## V. Discussion

The present study elucidates that the self-similarity and universality across different models in the hydrodynamic analog emerges as a result of the physics at small scales becoming so important, just as the universality in critical phenomena appears as a result of the physics at large scales becoming so important. Correspondingly, the self-similar solution naturally comes out as a fixed point of the RG transformation as in the case of critical phenomena. Although the original governing equation for the bubble breakup is a simple linear equation, the same self-similar solution is revealed to be universally shared by different equations, which could be very complex and nonlinear. All these features show close similarity with the counterpart in critical phenomena.

However, as seen above, there are crucial differences from the RG in critical phenomena. In the present case, there are no diverging characteristic lengths such as the correlation length. Instead, all the characteristic length scales go to zero as $t\rightarrow t_{c}$. Correspondingly, the coarse-graining process is replaced with magnification. This reflects the fact that the physics at large scales is important in critical phenomena, while that at small scales is important in the hydrodynamic analog. In addition, the observed master curve is identified as a stable fixed point. This is in contrast with the case of critical phenomena, where unstable fixed points are essential. This difference comes from the fact that in the hydrodynamic case the RG flow is considered for the space spanned by shape functions, not for the space spanned by parameters defining the Hamiltonian. Thus the observed master curve for the shape function is naturally interpreted as a stable fixed point (the observed shape is the one approached stably as $t\rightarrow t_{c}$), while in critical phenomena the unstable or relevant parameters determine the physics near the critical point.

Similarities and differences with the RG in critical phenomena discussed above can be explicitly seen in some expressions. For example, Eq. (13), $H_{L}(X,T)=L^{A}H(LX,L^{B}T)$, has a resemblance to the expression for the free energy $f_{s}(u_{t},u_{h})=b^{-d}f_{s}(b^{y_{t}}u_{t},b^{y_{h}}u_{h})$ in the notation of the textbook^30^, where *d* is is dimension and $y_{t}$ and $y_{h}$ are RG eigenvalues. Note that the scale factor *b* corresponds to *L*, from which we may regard $A=-d$, $B=y_{t}$, and $y_{h}=1$. However, as mentioned above, while $b>1$, $L<1$ in the present case. In addition, from the correspondence between Eq. (28), $H^{*}(X,T)=T^{-A/B}f^{*}(X/T^{1/B})$, with the re-expression of Eq. (5), $M=T^{(d-y_{h})/y_{t}}\Psi (H/T^{y_{h}/y_{t}})$, the dimension *A* may be identified with $-d+y_{h}$, rather than $-d$. We can understand these features as a result a deficiency of players in the hydrodynamic analog, with *h* playing multiple roles of the order parameter, free energy, and Hamiltonian.

The application of the present framework to recent example of bubble breakup in confined geometry^24,25,28,29^ is an important problem to understand deep connection between critical phenomena and self-similarity in partial differential equations. This issue will be discussed elsewhere in the near future.

## Data Availability

All data generated or analysed during this study are included in this published article
